# Non-Involuting Congenital Hepatic Hemangioma: Lessons from a Case Series

**DOI:** 10.3390/children12070893

**Published:** 2025-07-07

**Authors:** Karla Estefanía-Fernández, Paloma Triana, Carla Ramírez-Amorós, Mireia Gaspar-Pérez, Antonio Jesús Muñoz-Serrano, María Velayos, María San Basilio, Nelson M. Buitrago, Manuel Parrón, Ane Andrés, Francisco Hernández-Oliveros, Juan Carlos López Gutiérrez

**Affiliations:** 1 Department of Pediatric Surgery, La Paz Children’s University Hospital, 28046 Madrid, Spain; palomae.triana@salud.madrid.org (P.T.); antoniojesus.munoz@salud.madrid.org (A.J.M.-S.); maria.velayos@salud.madrid.org (M.V.); maricarmen.sanbasilio@salud.madrid.org (M.S.B.); ane.andres@salud.madrid.org (A.A.); fhernandezo@salud.madrid.org (F.H.-O.); queminfantil.hulp@salud.madrid.org (J.C.L.G.); 2 Department of Pediatric Surgery, University Hospital Parc Taulí, 08208 Sabadell, Spain; mgaspar@tauli.cat; 3 Department of Pediatric Radiology, La Paz Children’s University Hospital, 28046 Madrid, Spain; nelsonm.buitrago@salud.madrid.org (N.M.B.); manuel.parron@salud.madrid.org (M.P.)

**Keywords:** congenital hepatic hemangioma, hepatic vascular tumor, sirolimus, pediatric liver tumor, non-involuting hemangioma

## Abstract

**Background**: Congenital hepatic hemangiomas (CHHs) are typically considered rapidly involuting tumors, similar to their cutaneous counterparts (RICHs). However, non-involuting tumors remain poorly characterized. This study examines the evolutionary patterns and management strategies for non-involuting congenital hepatic hemangiomas (NICHHs). **Methods**: We conducted a retrospective review of clinical, imaging, histological, and genetic data of children diagnosed with NICHH—defined as showing no signs of involution for at least 18 months—between 1991 and 2022. **Results**: Seven patients (five females, two males) were identified. The median age at diagnosis was 42 days (range: 0–1440). Five patients had asymptomatic lesions, predominantly located in the right hepatic lobe. Histologic confirmation was available in three cases, and a *GNAQ* gene mutation was identified in one. The median follow-up period was 75 months (range: 35–191). Three patients with giant NICHH were treated with sirolimus, resulting in partial response in two cases and lesion stabilization in one. The four untreated patients showed diverse evolutionary patterns, including delayed involution and tardive growth. **Conclusions**: NICHH lesions demonstrate distinct long-term evolution. Accurate diagnosis and regular monitoring are essential to avoid unnecessary interventions. Sirolimus may offer a promising non-surgical treatment for select patients, particularly those with giant lesions.

## 1. Introduction

Congenital hepatic hemangiomas (CHHs) are benign, high-flow vascular tumors that develop in utero an are fully developed at birth [1]. While most are solitary and asymptomatic, a subset may cause significant complications such as intratumoral hemorrhage, thrombocytopenia, hypofibrinogenemia, or high output cardiac failure [1,2].

The classification and nomenclature of hepatic hemangiomas (HHs) has evolved over the years. In 2007, a systematic framework identified three types: focal, multifocal, and diffuse [3]. Subsequent validation by the Liver Hemangioma Registry at Boston Children’s Hospital confirmed that focal lesions represent CHH [4]. In 2018, the International Society for the Study of Vascular Anomalies (ISSVAs) classified HH into CHH and infantile hepatic hemangioma (IHH), based on clinical behavior and histopathological markers.

Initially, it was hypothesized that all CHH were rapidly involuting congenital hepatic hemangiomas (RICHHs), which regress within the first 12–18 months of life [4]. However, a subset of lesions with morphology consistent with CHH has been reported to minimal or no involution, even after extended follow-up beyond one year [2,4,5,6,7,8]. While cutaneous non-involuting congenital hemangiomas (NICHs), which have been extensively studied and described in the literature, their hepatic counterpart remain poorly characterized. This study presents a series of patients with NICHH, aiming to describe their clinical, radiological, histological, and genetic characteristics, and to evaluate the therapeutic role of sirolimus.

## 2. Materials and Methods

### 2.1. Data Collected

We performed a retrospective review of patients diagnosed with HH at our institution between 1991 and 2022. Patients diagnosed with CHH were further evaluated, and those with lesions persisting without signs of involution for at least 18 months were classified as NICHH. Data included demographic information, lesion characteristics (location and size), age at diagnosis, alpha-feto protein (AFP) levels, presence of cutaneous hemangiomas, clinical symptoms, imaging findings, histological and genetic analyses, follow-up duration, and treatments. For patients receiving sirolimus, additional data included dosage, blood levels, therapeutic response, and side effects.

### 2.2. Diagnosis, Follow-Up and Treatment

All patients underwent initial Doppler ultrasound. If the diagnosis remained uncertain, contrast-enhanced MRI was performed. Imaging was reviewed by a panel of pediatric radiologists who confirmed the absence of involution during the initial 18 months Patients were excluded if follow-up imaging was unavailable or spanned less than 18 months.

Follow-up included regular ultrasound examinations—monthly initially, then quarterly once stability was confirmed. In cases of lesion growth or diagnostic uncertainty, biopsy was performed to exclude malignancy and confirm diagnosis. Histological and genetic analyses were conducted on formalin-fixed, paraffin-embedded tissue using a 56-gene next-generation sequencing (NGS) panel and digital PCR for variant validation.

Given the evidence of positive response in patients with other vascular anomalies and similar mutations [9,10,11,12], treatment with sirolimus was offered to patients with giant NICHH (main diameter ≥ 4 cm) to reduce the necessity for surgical resection and to mitigate the risk of spontaneous and/or traumatic rupture. The parents and/or legal guardians of the all patients accepted treatment with sirolimus as compassionate use after being informed of the benefits, risks, and other therapeutic options. Dosing followed institutional guidelines: 0.8 mg/m^2^ every 12 h. Patients were monitored via ultrasound and MRI to assess response. Bloodwork was conducted every three months and included complete blood count, liver and renal function tests, lipid profile, and sirolimus levels.

### 2.3. Data Analysis

Descriptive analysis was performed using medians and ranges for continuous variables and percentages for categorical data. A literature review was conducted using PubMed, Google Scholar, and ClinicalTrials.gov with relevant terms in English and Spanish to identify previously reported cases of non-involuting hepatic hemangiomas.

## 3. Results

### 3.1. Demographic and Clinical Characteristics

A total of seven patients were included in this study. Their clinical and demographic characteristics are summarized in Table 1. There was a predominance of females (5 out of 7). Only two cases were diagnosed prenatally via US, and all were term newborns. Most patients were asymptomatic at birth; however, two presented with hepatomegaly and an abdominal mass, though without complications. AFP levels were normal in all patients, and none of the liver lesions were associated with cutaneous hemangiomas.

The majority of lesions were located in the right hepatic lobe (RHL) (five patients). Histological analysis was available for three patients, all of whom were diagnosed with CHH. Immunohistochemical staining revealed that the endothelial cells were positive for CD31 and CD34, but negative for Glut-1 and D2-40. The histology showed small vascular lobules surrounded by fibrous tissue, with no evidence of involution.

Genetic studies identified a pathogenic *GNAQ* variants: c.626A ≥ T; p.Gln209Leu.

### 3.2. Follow-Up and Radiological Characteristics

**Figure 1:**
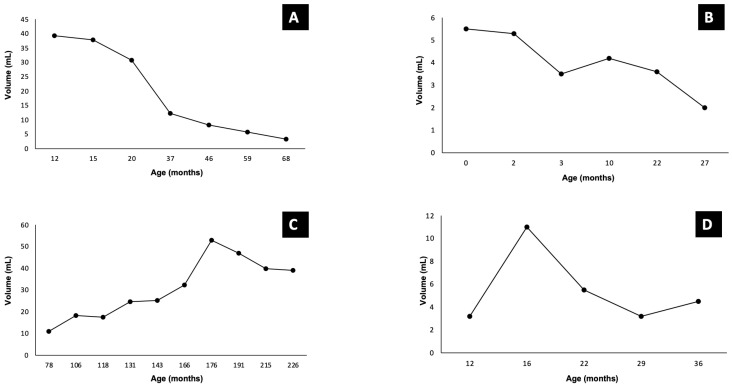
Time evolution curve of tumor volume in untreated patients (**A**–**D**).

The median follow-up period was 75 months (range: 35–191 months), and all patients were alive at the time of the last follow-up. Among the four patients who did not receive any medication, two showed delayed involution (Figure 1A,B), while the other two experienced late-phase growth (Figure 1C,D).

Ultrasound findings typically revealed solid tumors with poorly defined margins and multiple ill-defined hypoechogenic areas, corresponding to vascular structures confirmed via Doppler imaging. Gross calcifications were observed in many of the lesions.

MRI showed that all the lesions were hyperintense signal changes on T2-weighted images (T2W1) and hypointense on T1-weighted images (T1W1), with serpiginous signal voids consistent with intralesional vessels. A portosystemic shunt was identified in only one lesion, connecting the anterior branch of the right portal vein to a large draining vein that emptied into the middle hepatic vein.

Upon review of all imaging studies, no specific radiologic features were found to correlate with the lesion’s evolution pattern in NICHH.

### 3.3. Treatment and Sirolimus

Three patients with lesions larger than 4 cm and without signs of involution were treated with sirolimus. Data of patient treatment and outcome are summarized in Table 2.

Sirolimus therapy was initiated at a median age of 30 months (range: 12–49 months) and continued for a median duration of 39 months (range: 34–45 months). Therapeutic drug monitoring was conducted for all patients, maintaining sirolimus blood levels within the target range of 4 and 12 ng/mL. No adverse effects were reported except for a single case of self-limiting mucositis, and no dose adjustments were required.

**Figure 2:**
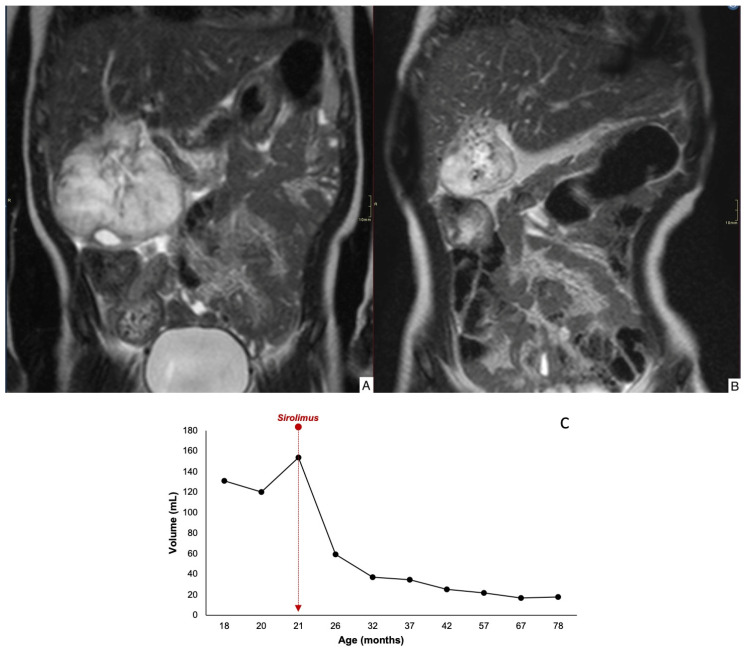
Coronal T2-weighted MRI image (T2WI) through the abdomen of an 1-yr-old girl depicts an exophytic and T2-hyperintense mass in the liver (**A**). Coronal T2WI after 18 months of treatment with sirolimus, the mass has markedly decreased in size (84% tumor volume reduction) (**B**). Time evolution curve of tumor volume (**C**).

**Figure 3:**
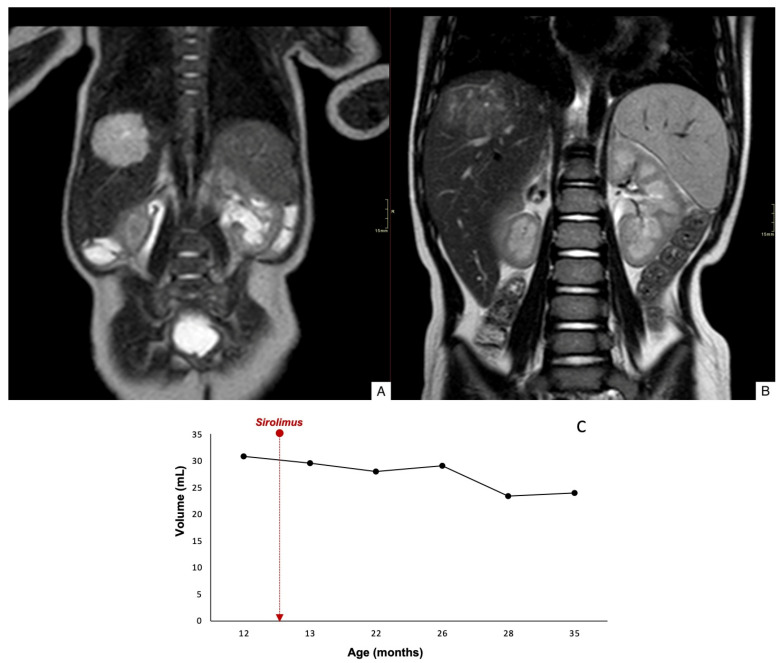
Coronal T2WI through the abdomen of a 1-mo-old boy shows a hyperintense lesion in the right lobe of the liver (**A**). Coronal T2WI after 18 months of treatment with sirolimus, the lesion size is stable, but with decreased T2-signal intensity (**B**). Time evolution curve of tumor volume (**C**).

**Figure 4:**
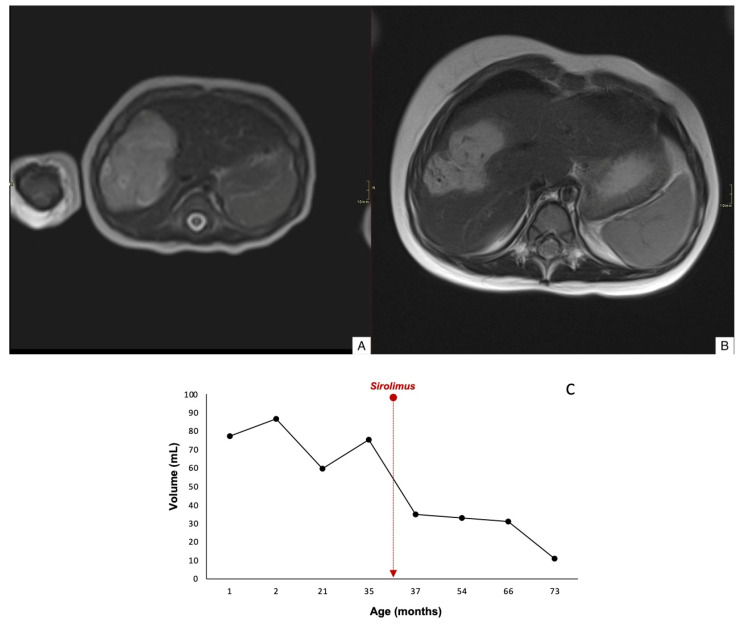
Axial T2WI through the liver of a 1-month-old girl (case 3) revealing a large hypertense mass in the right lobe of the liver (**A**). Axial T2WI after 18 months of treatment with sirolimus, the lesion shows a 60% tumor volume reduction with persistent portosystemic venous fistulas (appear as serpiginous signal voids within the mass) (**B**). Time evolution curve of tumor volume (**C**).

Case 1 demonstrated an 84% reduction in lesion volume, along with decreased peripheral contrast enhancement and complete regression by 78 months of age (Figure 2). Case 2 showed a mild reduction in lesion size, with decreased uptake and worsening margin definition; the lesion remained stable at 35 months of age (Figure 3). Case 3 exhibited an 60% reduction in tumor volume, although complete involution did not occur, and portosystemic fistulas persisted (Figure 4).

After one year of sustained positive response and lesion stability, a gradual tapering of sirolimus was initiated in Case 1. Following confirmation of lesion stability and absence of rebound growth, the therapy was successfully discontinued. The other two patients continue to receive sirolimus at the time of this report.

## 4. Discussion

This study presents one of the largest case series to date focused on NICHH, confirming their existence and highlighting their unique evolutionary patters. While CHH have traditionally been considered to parallel the behavior of cutaneous congenital hemangiomas, our findings suggest otherwise [4,5,7]. Only a few cases have been documented in the literature as NICHH. In 2012, Kulungowski et al. [4] reported a focal HH with no change after three years of follow-up. Other reviews have similarly identified patients with CHH who showed no signs of evolution: one study reported two patients without radiological changes after 24 and 72 months, another documented two cases of 13 patients with no evidence of regression [5,6,7]. Based on these reports and the involution patterns of their cutaneous counterparts, we identified seven patients with NICHH in our cohort—all of whom showed no change or involution during the first 18 months of follow-up.

Unlike cutaneous NICH, which exhibit an equal sex distribution and typical anatomic predilection [13], our NICHH patients were predominantly female, with a higher frequency of tumors affecting the RHL. Given their presentation as large hepatic masses, the differential diagnosis includes hepatoblastoma, mesenchymal hamartoma, and metastatic tumors in children. While normal AFP levels can help differentiate benign from malignant lesions, AFP may remain elevated in CHH [14,15]. In this series, AFP levels were normal, but three patients underwent biopsy due to diagnostic uncertainty based on imaging. Histological confirmation was achieved in these cases, and the *GNAQ:* p.Gln209Leu variant—previously described by our group—was identified in one patient [7].

There is currently no consensus regarding optimal follow-up imaging protocols for CHH. Nonetheless, existing guidelines emphasize the importance of stablishing the lesion’s evolutionary pattern. RICHH typically regress by about 80% by 12–13 months of age, while NICHH remain stable, similar to cutaneous hemangioma [14,16]. Notably, our cohort revealed variability in lesion behavior: two untreated patients exhibited delayed regression, possibly representing a Partially Involuting Congenital Hepatic Hemangioma (PICHH)—a subtype not yet described in hepatic lesions. Conversely, two patients demonstrated late-onset growth (tardive expansion), resembling a recently defined cutaneous hemangioma entity known as Tardive Expansion Congenital Hemangioma (TECH) [8]. While the cause of the expansion remains unclear, we suggest that in such cases, TECH should be considered, and malignancy ruled out via biopsy before contemplating liver resection. These findings underscore the critical role of long-term imaging in accurately classifying CHH and guiding treatment decisions.

Guidelines for CHH treatment remain limited. It is well-established that asymptomatic patients should undergo observation and close surveillance [14]. In symptomatic cases with severe complications, giant lesions, lack of response to drug therapy, or failure to involution, embolization or surgical resection may be warranted. Although tumor size is not consistently cited as a predictor of mortality [17], some studies have reported resections for CHH larger than 4 cm, often due to suspicion of malignancy or lack of involution [5,6]. The median age of patients undergoing resection was typically under one year, suggesting that many of these tumors may have followed a NICHH pattern—and potentially could have avoided surgery and its associated risks.

In adult patients with HH, Mocchegiani et al. [18] reported a spontaneous rupture rate of 0.47% in a cohort of 2071 patients, with mortality rate of 20% among cases presenting with hemoperitoneum. Their recommended surgical intervention for giant, peripherally located hemangiomas with exophytic growth. Conversely, Donati et al. [19] emphasized the rarity of spontaneous rupture and suggested reserving surgery for lesions larger than 11 cm. In our NICHH series, none of the patients experienced complications during the neonatal period or during follow-up, further supporting the indolent nature of these tumors.

There is limited evidence supporting the effectiveness of pharmacological treatment for CHH. While propranolol, corticosteroids, and vincristine have been used in infantile hemangiomas, their efficacy in focal congenital hemangiomas remains unproven. However, based on previous successful experiences with sirolimus in treating other vascular conditions associated with *GNAQ* mutation—as well as in neonates with complicated CHH—and our clinical experience, we initiated sirolimus therapy in three patients with NICHH [9,10,11,12,20,21,22,23]. Two patients demonstrated a >50% reduction in lesion size over 15 months of treatment, while a third showed a mild response. These findings suggest that sirolimus may help reduce lesion size or accelerate involution. We recommend considering pharmacological treatment in NICHH cases with lesions > 4 cm to reduce the risk of traumatic rupture or avoid surgery.

The inconsistent use of the term “hemangioma” and the lack of comprehensive data on NICHH have hindered clear characterization and registry of these lesions. We identified 29 reported cases of focal hepatic lesions persisting beyond 24 months [2,4,5,6,7,24,25,26,27]. Although most lacked histologic confirmation, we believe they likely represent NICHH. Furthermore, our review of adult hepatic vascular tumors revealed the recently described *Hepatic Small Vessel Neoplasm* (HSVN), a vasoformative tumor of uncertain malignant potential with GNAQ mutations similar to CHH [28,29,30,31]. This molecular similarity may indicate a shared pathogenesis. Most of our patients were diagnosed incidentally in childhood, suggesting that undiagnosed NICHH may persist into adulthood and potentially be misclassified as HSVN. Therefore, further genetic and histologic comparisons between NICHH and HSVN are warranted to clarify their relationship and determine whether they represent a spectrum of the same neoplastic process.

## 5. Limitations

The main limitation of this study is its retrospective design, which may introduce selection and information bias. Additionally, the small sample size limits our ability to draw definitive conclusions regarding the efficacy of sirolimus in NICHH. However, given the rarity of NICHH and the extended follow-up period in our cohort, this study provides valuable insights into the natural history and distinct behavior of these lesions.

## 6. Conclusions

This series confirms the existence of NICHH and emphasizes the variability in CHH evolution. Long-term imaging follow-up is essential for accurate classification and appropriate management. In selected cases, sirolimus may offer a non-surgical therapeutic option, potentially reducing the need for liver resection. Further studies with larger cohorts are needed to validate these findings.

## Figures and Tables

**Table 1:** Patient characteristics.

Demographics and Clinical Characteristics	Study Cohort (n = 7)
Gender	
Female	5/7
Male	2/7
Age at diagnosis	
Prenatal diagnosis	2/7
Postnatal diagnosis	5/7
Gestational age	38 weeks (38–40)
Age at diagnosis	42 days (0–1440)
Clinical features	
Asymptomatic	5/7
Symptomatic	2/7
AFP value	
Normal	7/7
Elevated	0/7
The site in the liver	
Right lobe	5/7
Left lobe	2/7
Liver biopsy	
Yes	3/7
No	4/7

**Table 2:** Patient Treatment and Outcome.

Case	Maximum Diameter of Mass (cm)	Time of Intervention (Months)	Overall Response	Radiological Response (% of Reduction)	Duration of Treatment (Months)	Adverse Effects or Complications	Ongoing Treatment	Other Concomitant Treatments	Follow up (Months)
1	7 × 6 × 6 >4 cm	21	Partial	84%	69	No	Yes	Propranolol	79
2	3.7 × 4.6 × 3.4 >4 cm	12	Stable	Stable	45	No	Yes	No	56
3	5 × 3.5 × 6 >4 cm	36	Partial	60%	34	No	Yes	No	81

## Data Availability

The data that support the findings of this study are available from the corresponding author upon reasonable request, as they contain Hospital IDs and other personal information of the patients.
